# Supplementary material for: Robust LC3B lipidation analysis by precisely adjusting autophagic flux
Source: Sci Rep. 2022 Jan 7;12:79. doi: 10.1038/s41598-021-03875-8 (PMC8742033; doi:10.1038/s41598-021-03875-8)

Robust LC3B lipidation analysis by precisely adjusting autophagic flux

Martina P. Liebl, Sarah C. Meister, Lisa Frey, Kristina Hendrich, Anja Klemmer, Bettina Wohlfart, Christopher Untucht, Judith Nuber, Christian Pohl, Viktor Lakics

Supplementary information

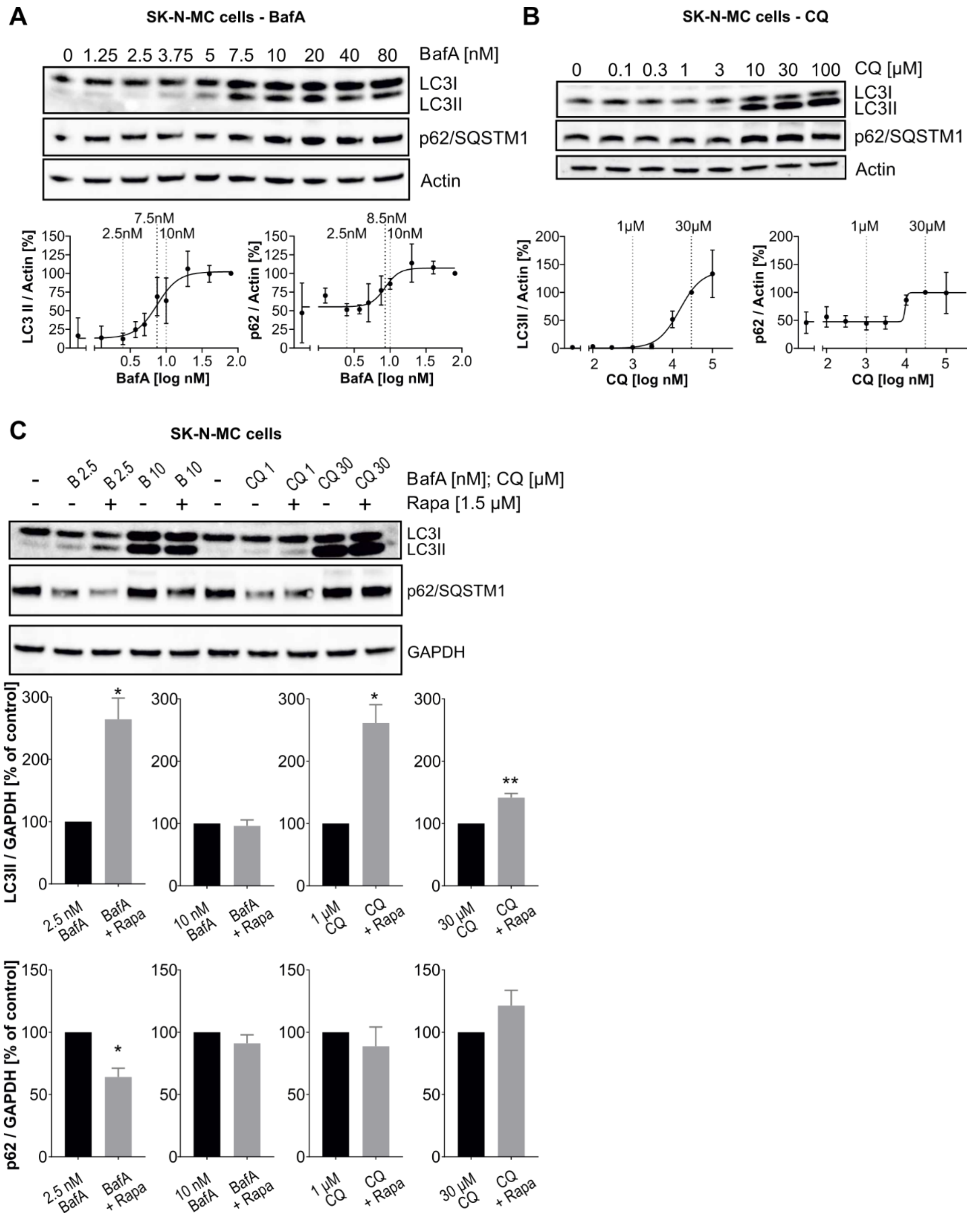

Supplementary Figure 1. Applying non-saturating and saturating concentrations of late-stage autophagy inhibitors to SK-N-MC cells. (**A**) SK-N-MC cells were treated with a concentration range of BafA (1.25 nM – 80 nM) for 24 h. Shown is a representative Western blot for LC3, p62/SQSMT1 and actin (top) as well as quantification based on Western blot analysis from 3 independent biological replicates (n = 3; mean +/- SEM). BafA inhibited the basal autophagic flux with an EC_{50} of 7.5 and 8.5 nM based on the increases of LC3-II and p62/SQSTM1, respectively. For curve display, LC3-II or p62/SQSTM1 levels at 80 nM BafA were set to 100 %. For curve fitting, a non-linear regression curve fit (log(inhibitor) vs. response- variable slope (four parameters)) was applied. (**B**) SK-N-MC cells were treated with a concentration range (0.1 – 100 µM) of CQ for 24 h. Shown is a representative Western blot for LC3, p62/SQSMT1 and actin (top) as well as quantification based on Western blot analysis from 3 independent biological replicates (n = 3; mean +/- SEM). The calculated EC_{50}s with

respect to LC3-II increase as well as p62/SQSTM1 accumulation were 15 µM and 9 µM, respectively. Curve calculation was done based on 3 independent biological replicates (mean +/- SEM). For curve display, LC3-II or p62/SQSTM1 levels at 30 µM CQ were set to 100 %. For curve fitting, a non-linear regression curve fit (log(inhibitor) vs. response- variable slope (four parameters)) was applied. **(C)** SK-N-MC cells were cotreated with either 2.5 nM or 10 nM BafA or either 1 µM or 30 µM CQ and 1.5 µM Rapamycin for 24 h. Shown is a representative Western blot for LC3, p62/SQSTM1 and GAPDH (left) as well as the quantification (right) from Western blots from 4 independent biological replicates (n = 4; mean +/- SEM). Statistics: One-sample t-test; compared to 100 % control (late-stage autophagy inhibitor only control, from left to right: 2.5 nM BafA, 10 nM BafA, 1 µM CQ, 30 µM CQ).

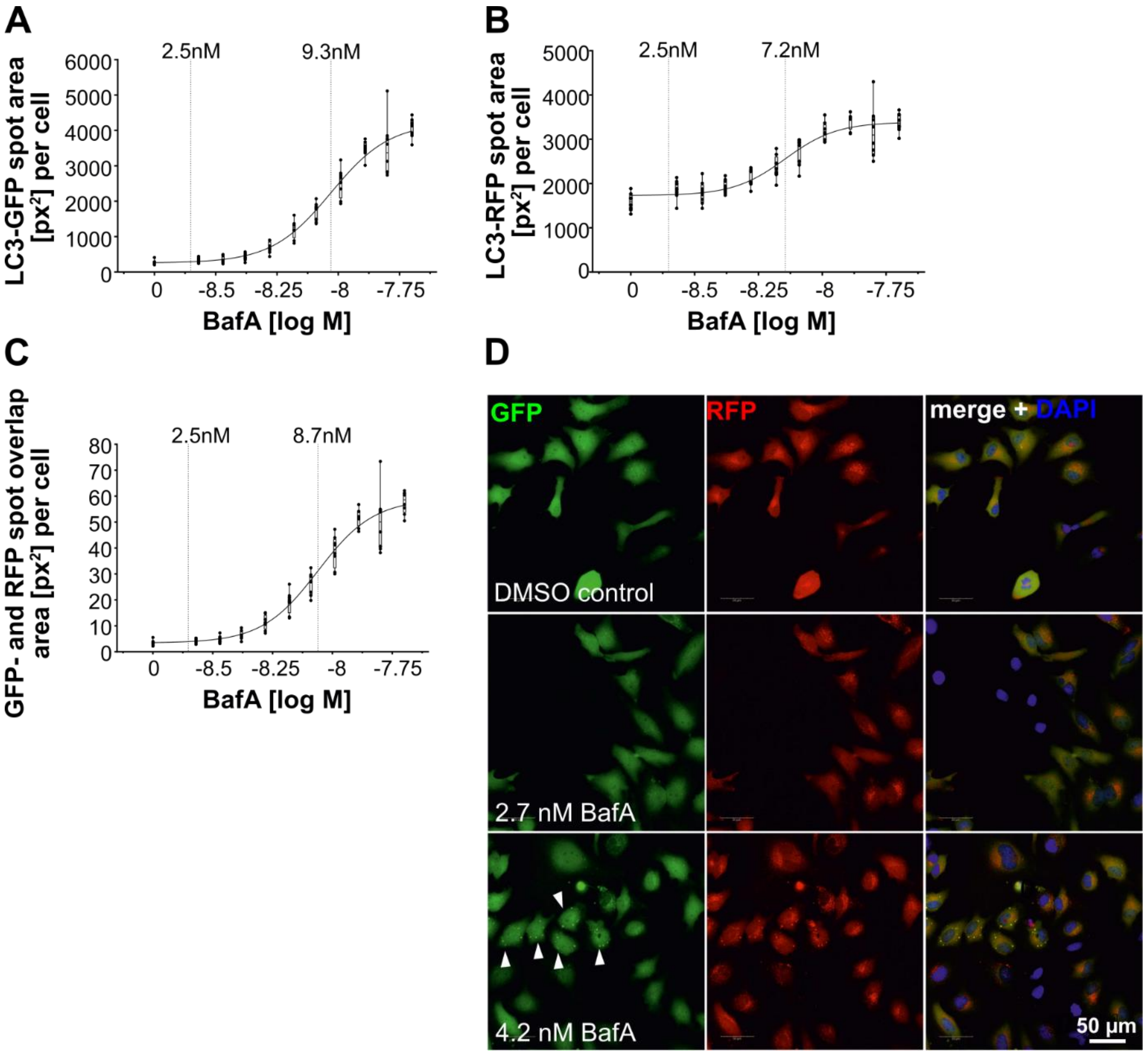

Supplementary Figure 2. Titration of BafA in HeLa GFP-RFP-LC3B Difluo cells and evaluation by fluorescence microscopy. (A) Quantification of GFP spot area, **(B)** RFP spot area and **(C)** co-localization of GFP and RFP positive spots for a concentration range of BafA (n = 8). **(D)** Representative fluorescence images from treatments quantified in panels (A) and (B). Only concentrations around the “non-saturating” concentration of BafA are shown. Arrowheads in the micrographs from 4.2 nM BafA treatments point to cells with LC3B puncta. For curve fitting, a non-linear regression curve fit (sigmoidal, 4PL, X is log (concentration)) was applied.

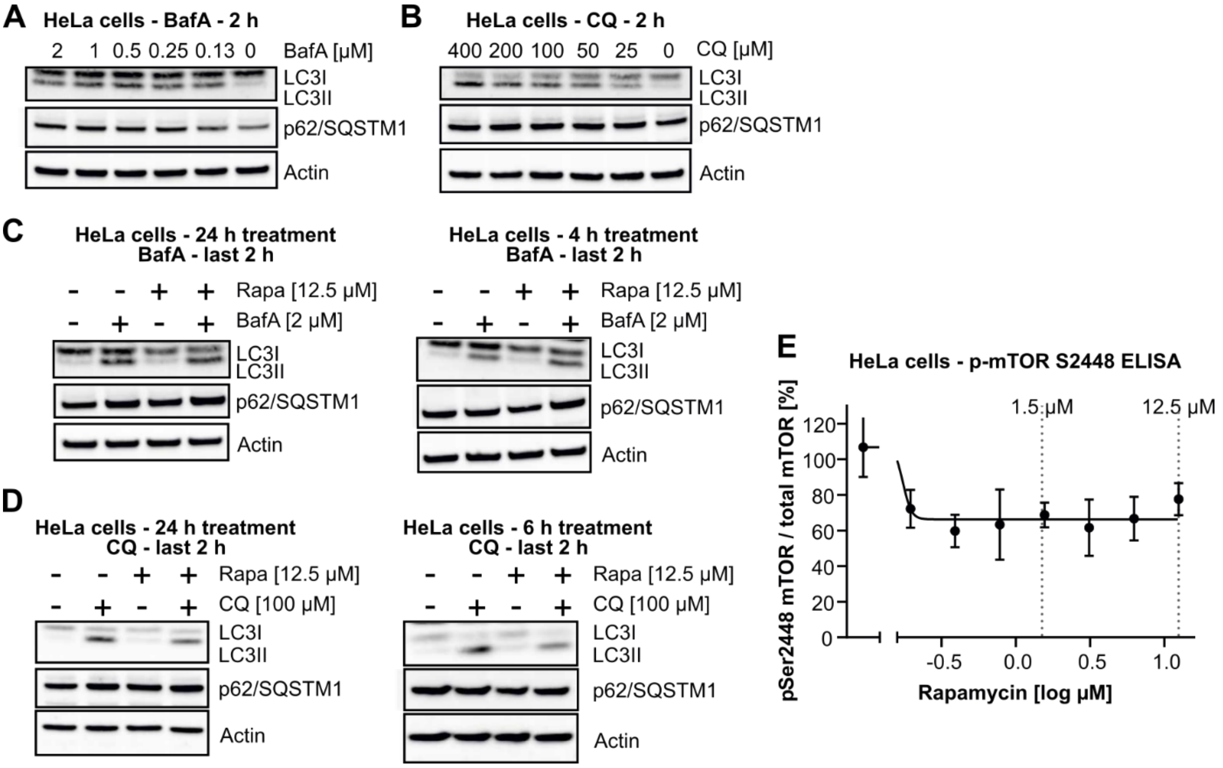

Supplementary Figure 3. Short-time treatments of HeLa cells with late-stage autophagy inhibitors and mTOR ELISA assay. For all experiments, representative Western blots for LC3, p62/SQSTM1 and actin are shown. **(A)** HeLa cells were treated with a concentration range (130 nM – 2 µM) of BafA for 2 h to determine a saturating concentration based on the LC3-II and p62/SQSTM1 increase. **(B)** HeLa cells were treated with a concentration range (25 µM – 400 µM) of CQ for 2 h to determine a saturating concentration based on the LC3-II and p62/SQSTM1 increase. **(C)** HeLa cells were treated with rapamycin for either 24 (left) or 4 h (right). BafA at a saturating concentration (2 µM) was added during the last 2 h of incubation with rapamycin. **(D)** HeLa cells were treated with rapamycin for either 24 (left) or 6 h (right). A saturating concentration of CQ (100 µM) was added during the last 2 h of incubation with rapamycin. **(E)** Lysates from HeLa cells treated with a dose range of rapamycin (200 nM – 12.5 µM) for 24 h were analyzed with a mTOR MSD ELISA assay multiplexing total mTOR and pS2448 mTOR. A maximum reduction of mTOR phosphorylation by 30-40 % was observed. The curve represents one biological replicate with 3 technical replicates (n=1; technical mean +/- SEM). For curve fitting, a nonlinear regression curve fit was applied (sigmoidal, four parameter logistic, X is log(concentration)).

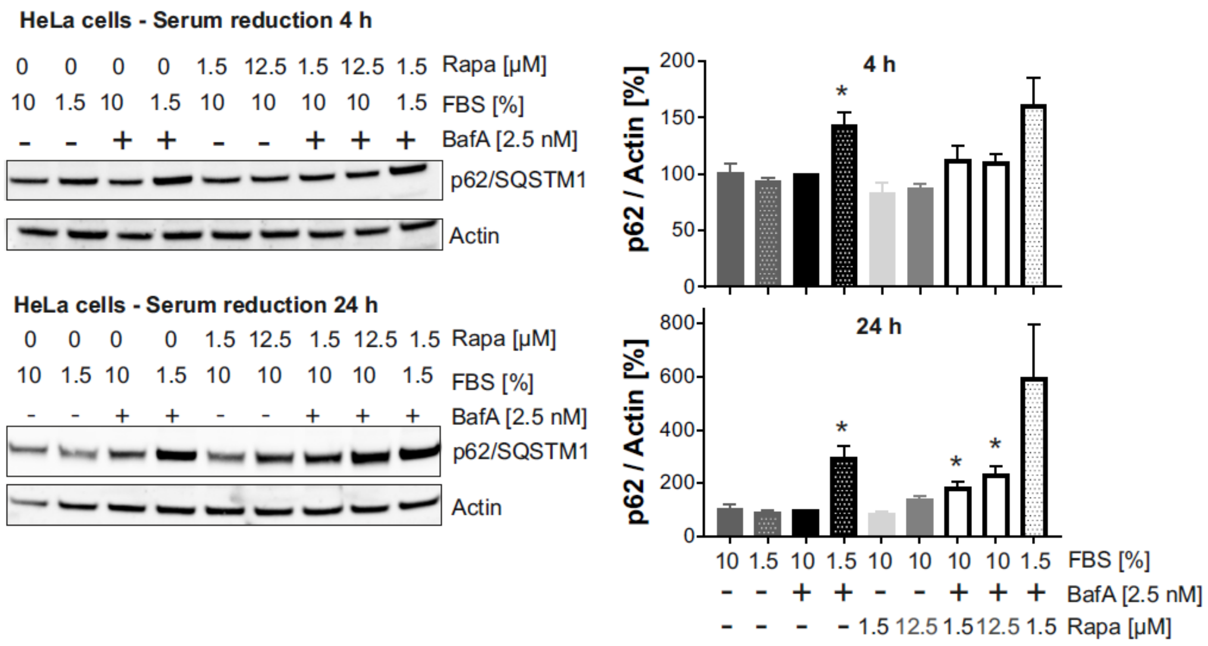

Supplementary Figure 4. Capturing autophagic flux increase through serum starvation by applying a non-saturating concentration of BafA. HeLa cells were kept in standard medium (10 % FBS) or in FBS-reduced medium (1.5 %) for 4 or 24 h. Medium change was combined with (co)-treatment of cells with 2.5 nM BafA and / or 1.5 or 12.5 µM rapamycin. Shown are representative Western blots of p62/SQSTM1 and actin (top) as well as quantifications (n=3, mean +/- SEM; bottom). Statistics: One-sample T-test; compared to 100 % control (DMSO, 10% FBS).

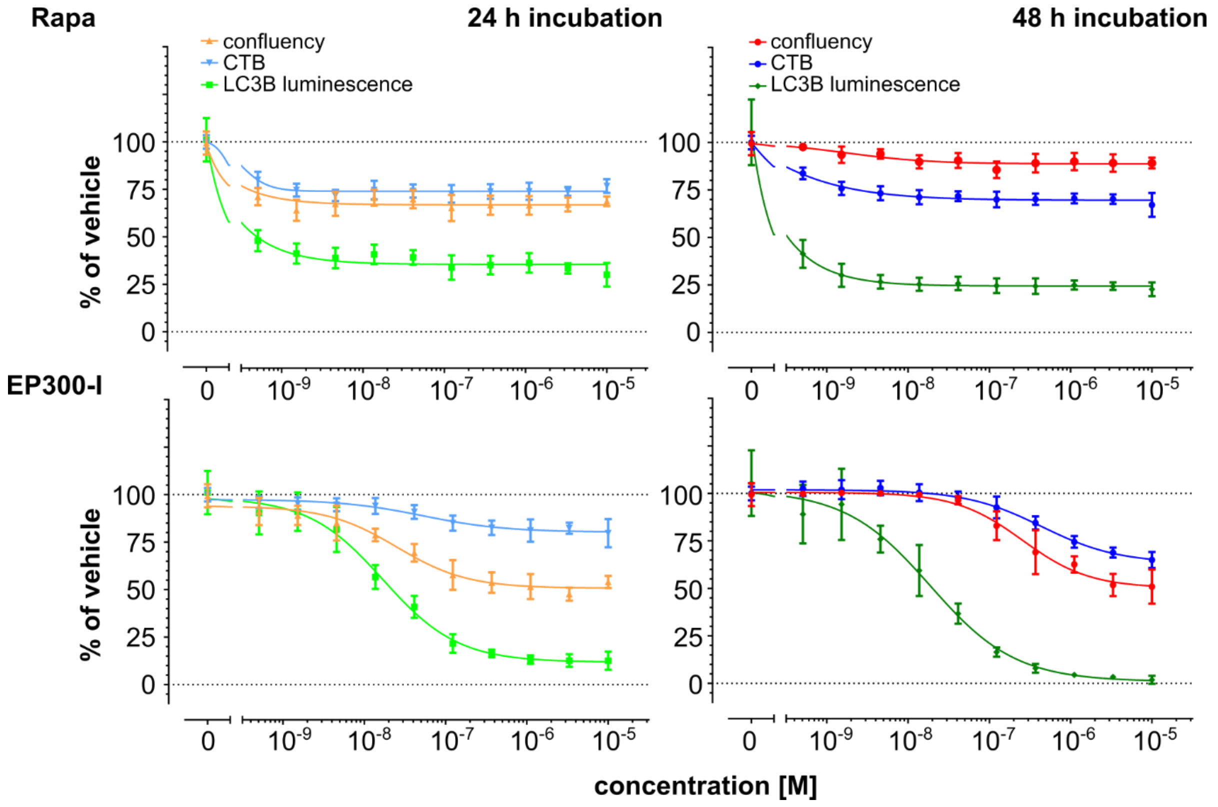

Supplementary Figure 5. LC3B luminescence complementation assay comparing concentration ranges of rapamycin to EP300-I. Measurement of confluency (orange/red), Cell Titer Blue (CTB, light and dark blue) and LC3B fluorescence complementation (light and dark green) for cells treated with different concentrations of rapamycin (top) and the EP300 inhibitor (bottom) for 24 and 48 h, respectively (n = 8).

Uncropped Western Blots

Figure 1A

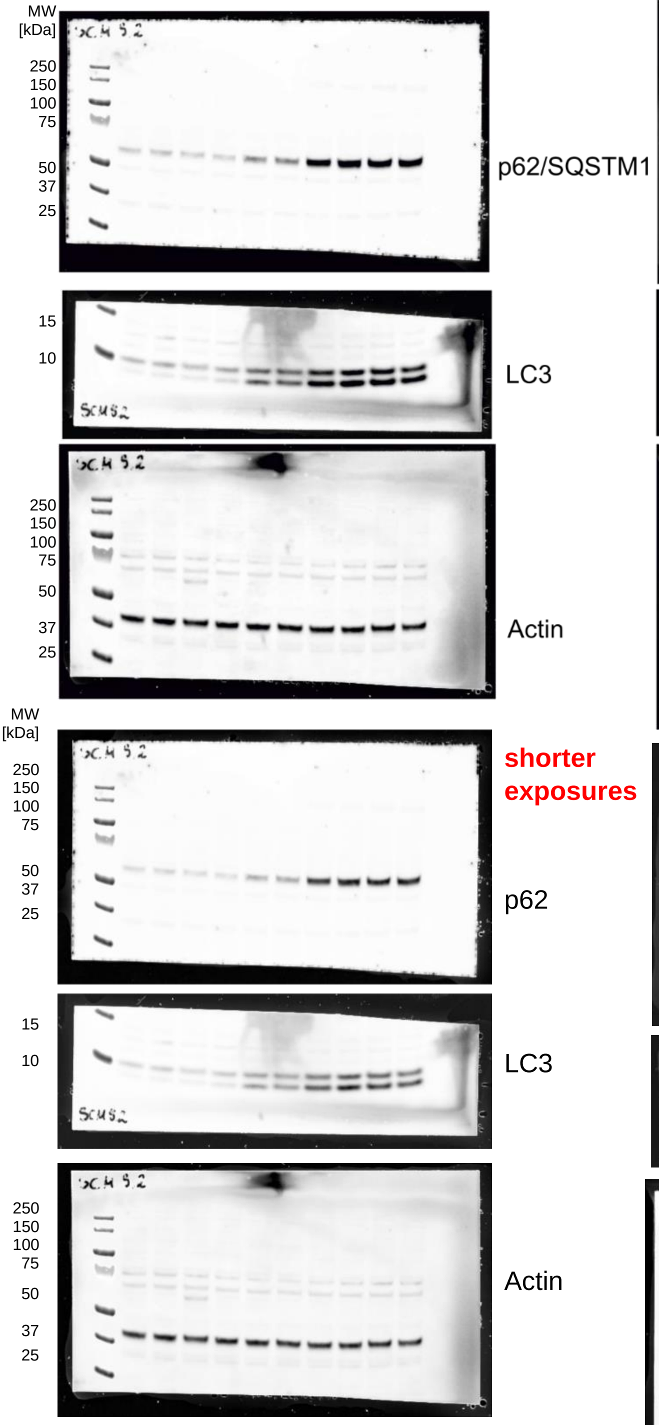

Figure 1B

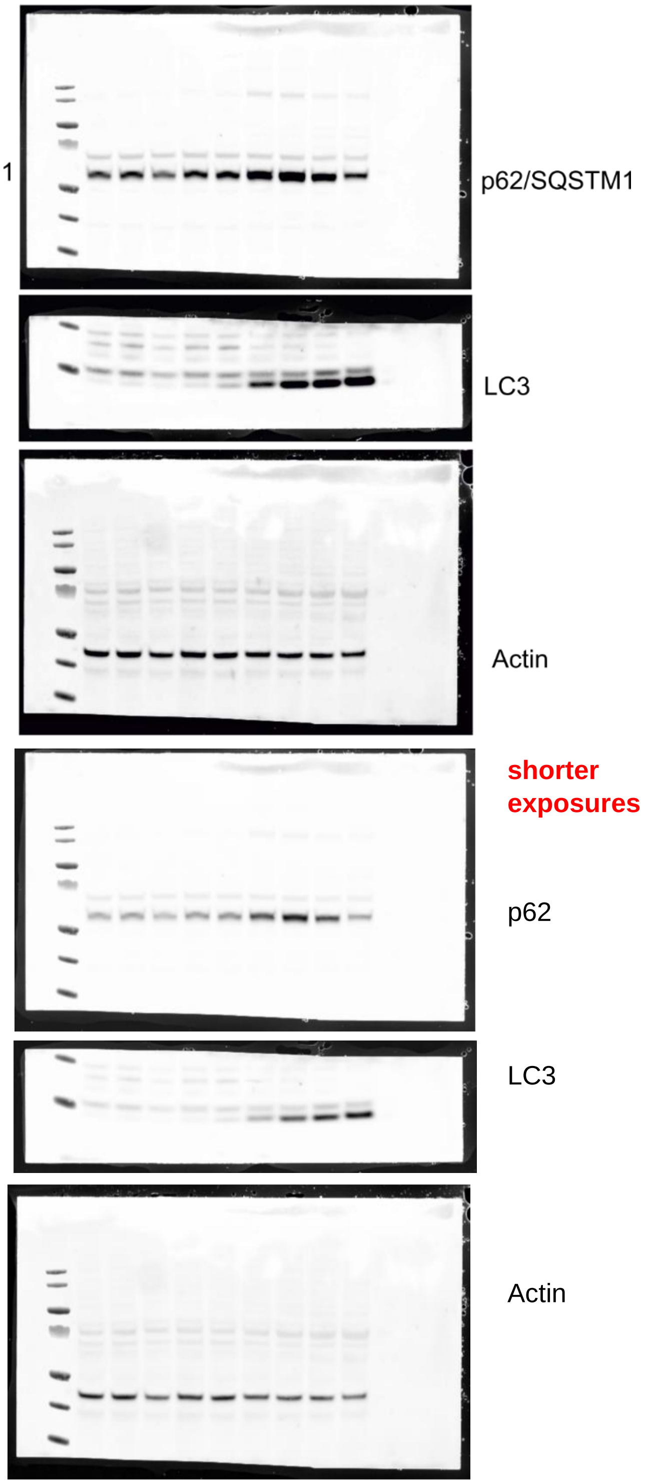

Figure 2A

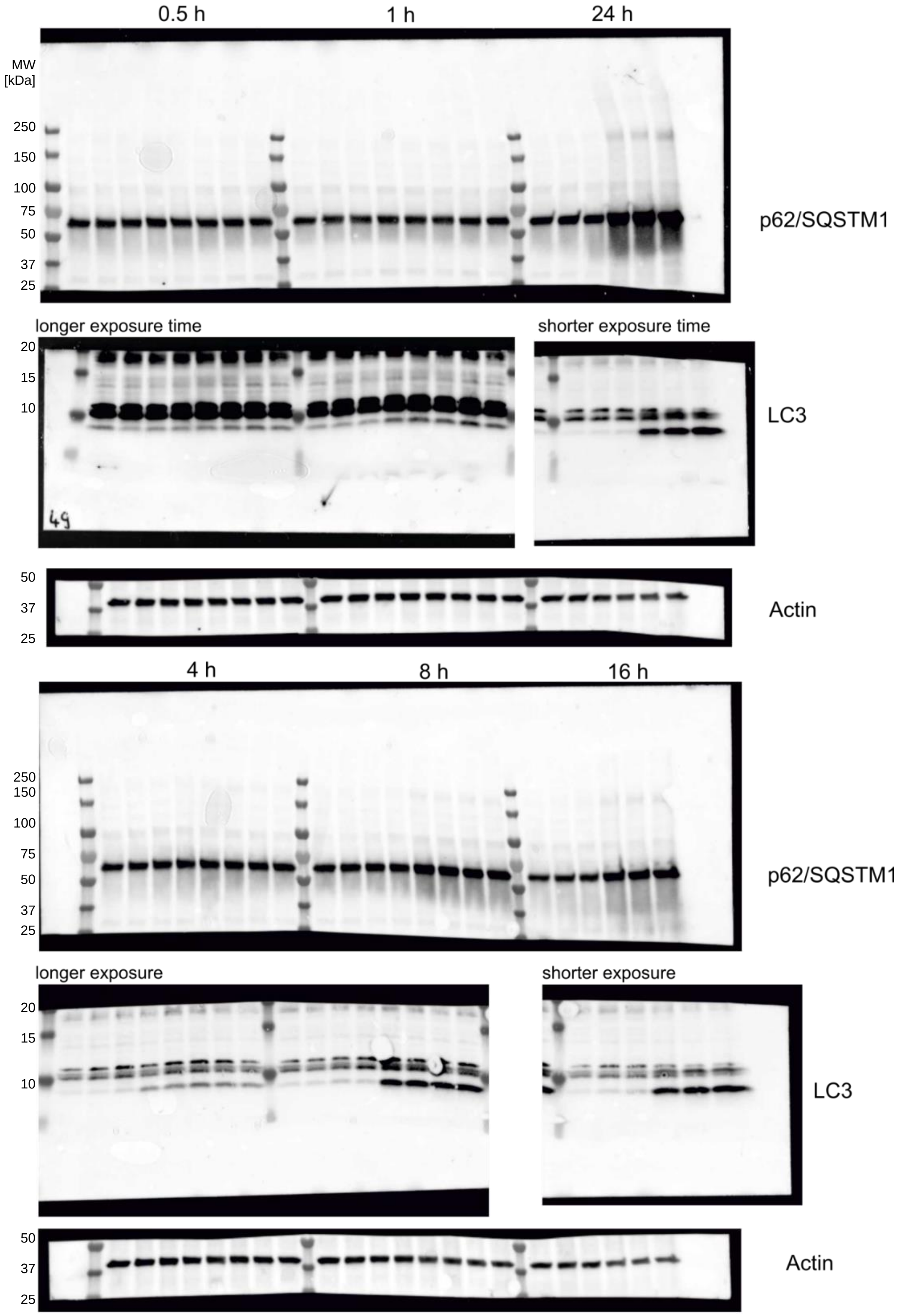
0.5 h
1 h
24 h
MW [kDa]
250
150
100
75
50
37
25
p62/SQSTM1
longer exposure time
shorter exposure time
20
15
10
LC3
50
37
25
Actin
4 h
8 h
16 h
250
150
100
75
50
37
25
p62/SQSTM1
longer exposure
shorter exposure
20
15
10
LC3
50
37
25
Actin

Figure 3A

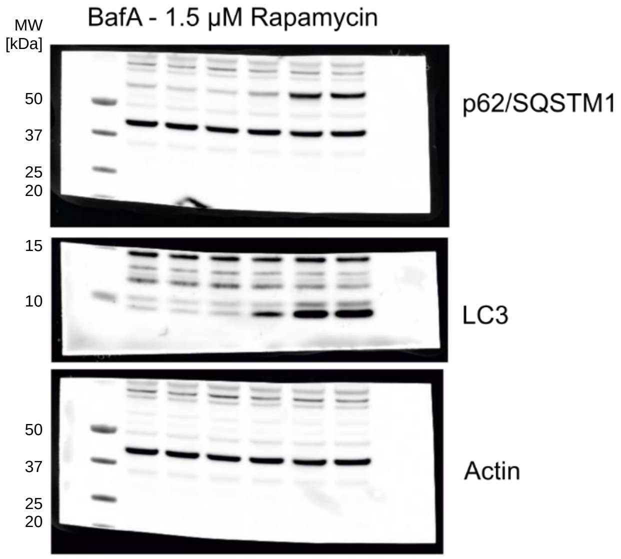

Figure 3C

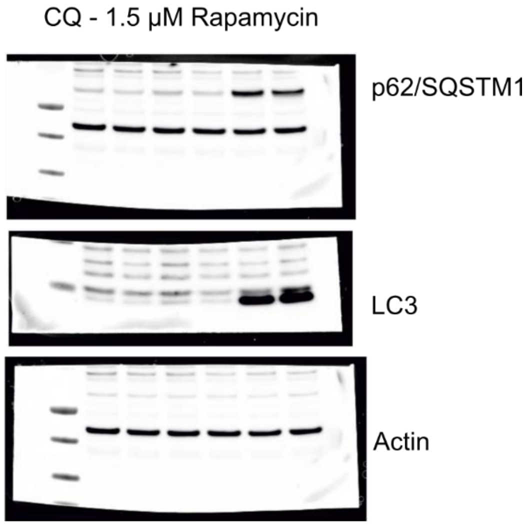

Figure 3B **Figure 3C**

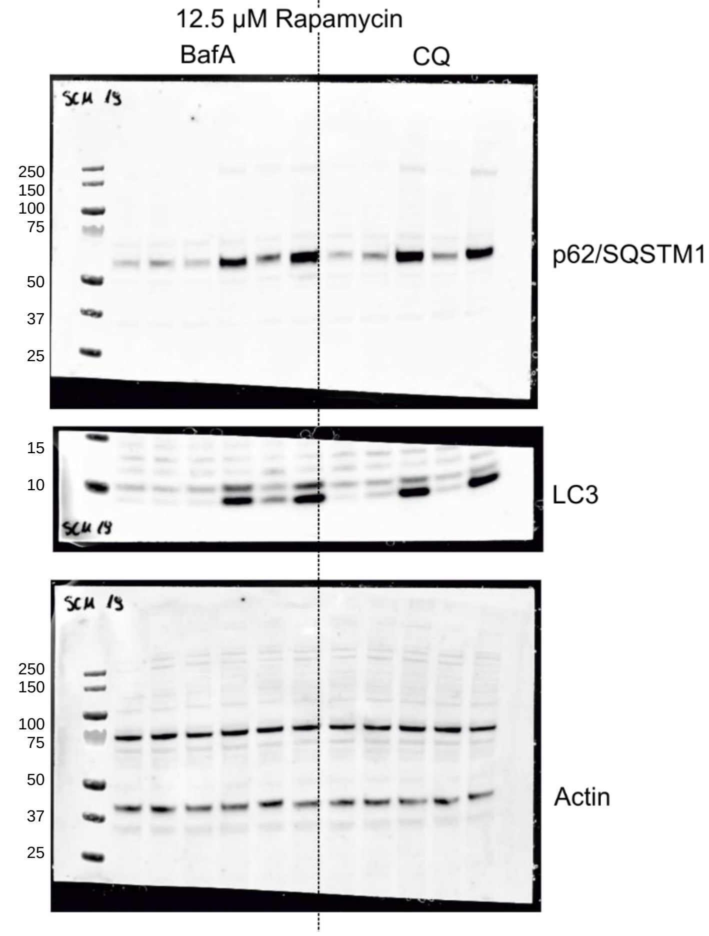

Figure 3A

BafA - 1.5 μM Rapamycin

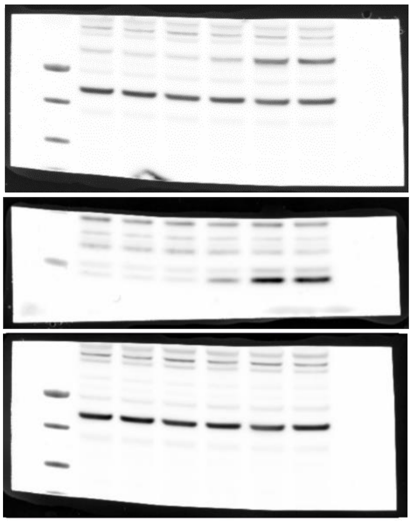

Figure 3C **shorter exposures**

CQ - 1.5 μM Rapamycin

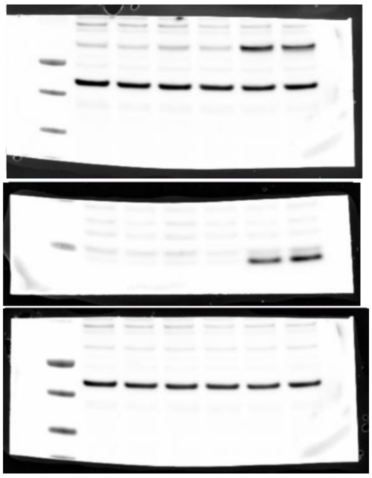

Figure 3B | **Figure 3C**

12.5 μM Rapamycin

BafA | CQ

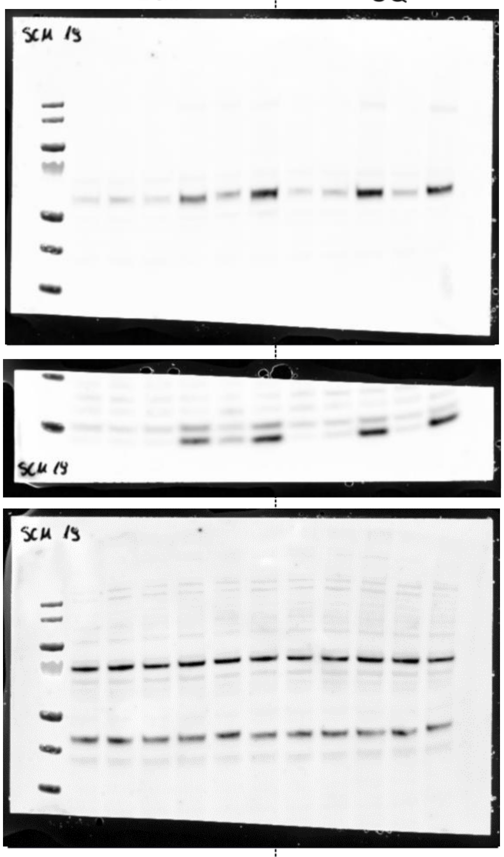

Figure 4A **Figure 4B**

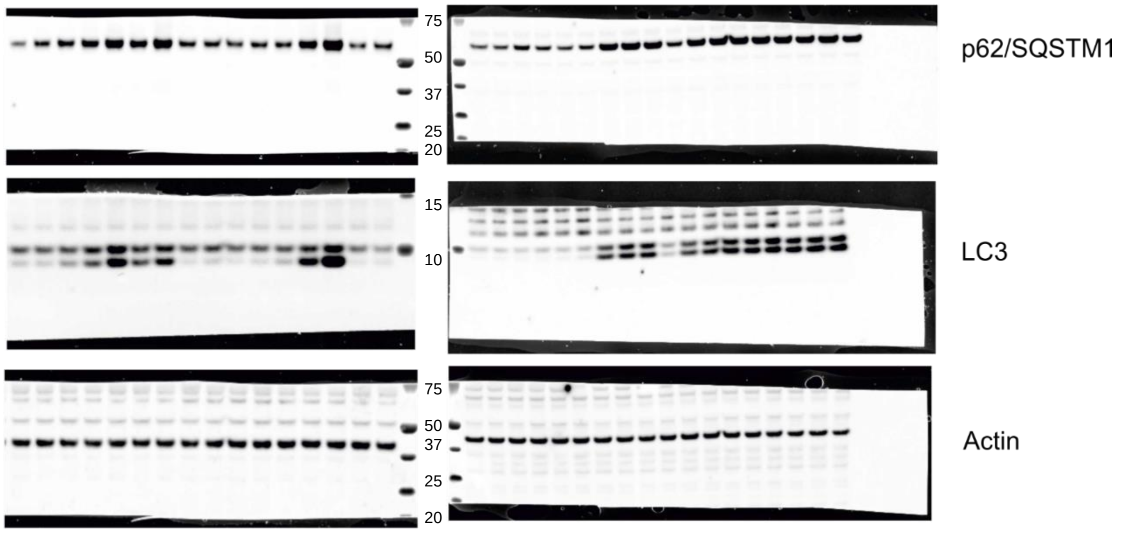

Figure 4A **Figure 4B** shorter exposures

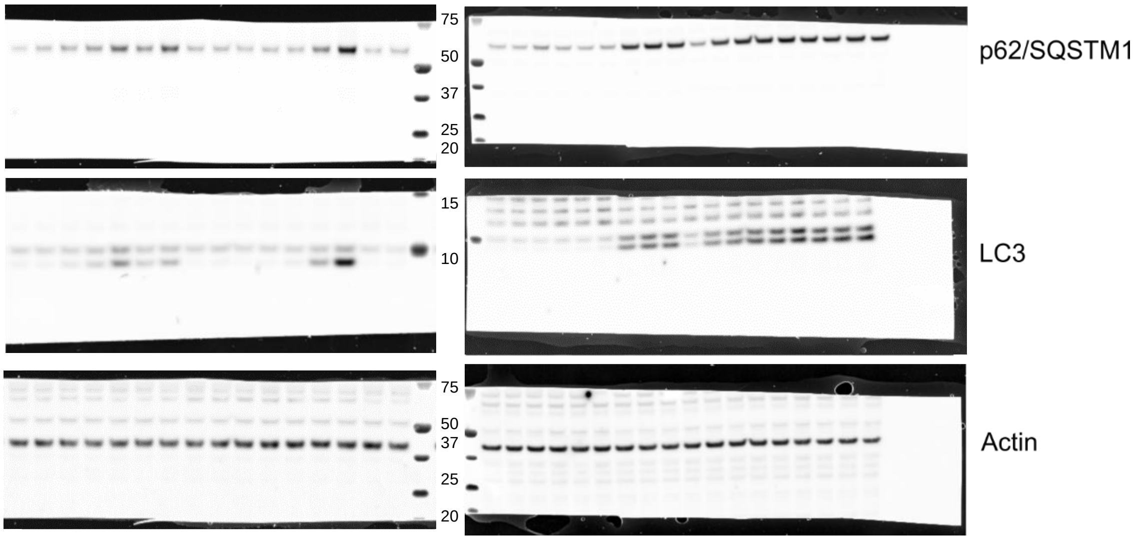

Figure 5A and Supplementary Figure 4

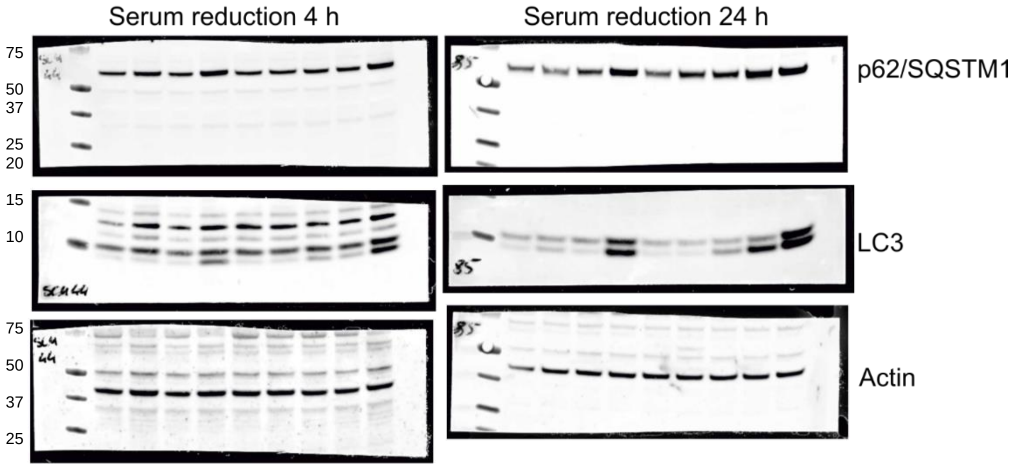

Figure 5B

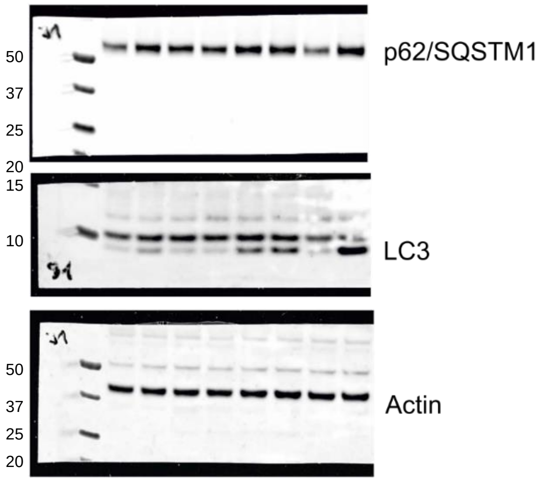

Figure 5A and Supplementary Figure 4 **shorter exposures**

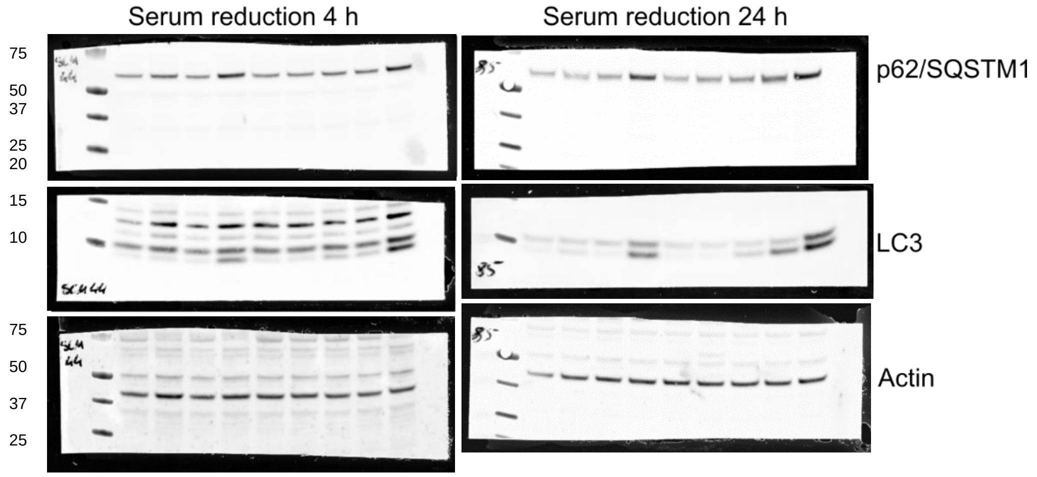

Figure 5B

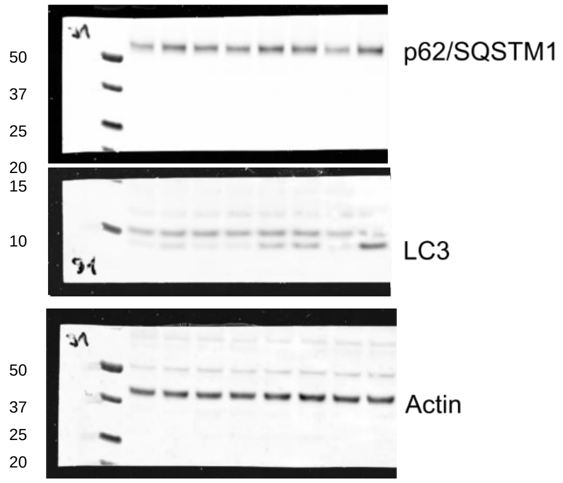

Figure 6 B

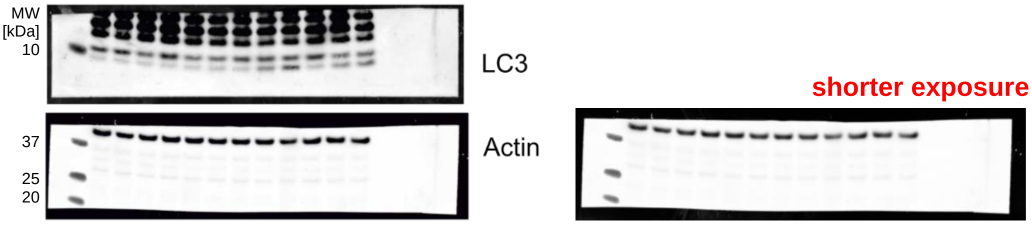

Figure 6 C

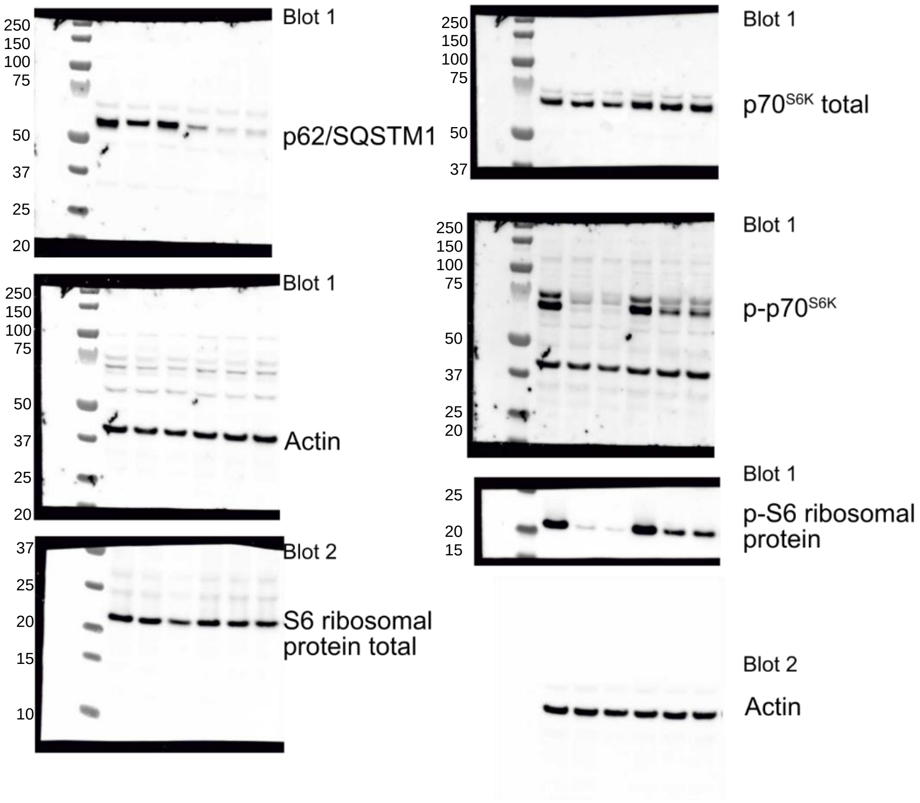

Figure 6 C

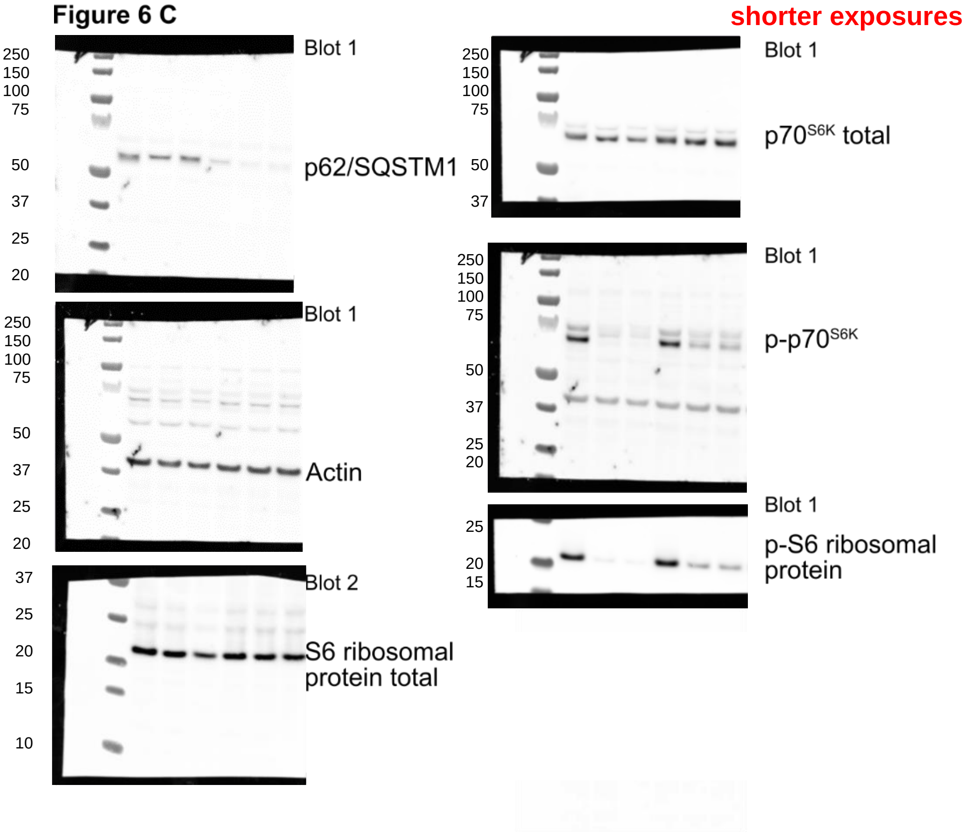
shorter exposures
Blot 1
p62/SQSTM1
250
150
100
75
50
37
25
20
Blot 1
Actin
Blot 2
S6 ribosomal protein total
10
15
Blot 1
p70^S6K total
Blot 1
p-p70^S6K
Blot 1
p-S6 ribosomal protein

Supp. Fig. 1A

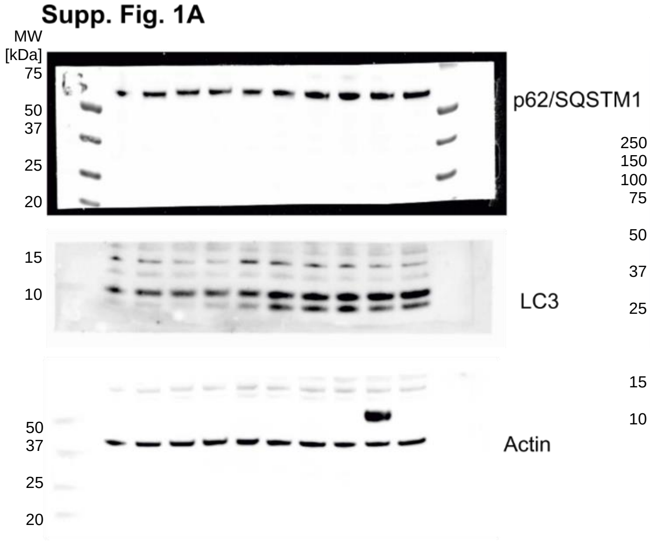

Supp. Fig. 1B

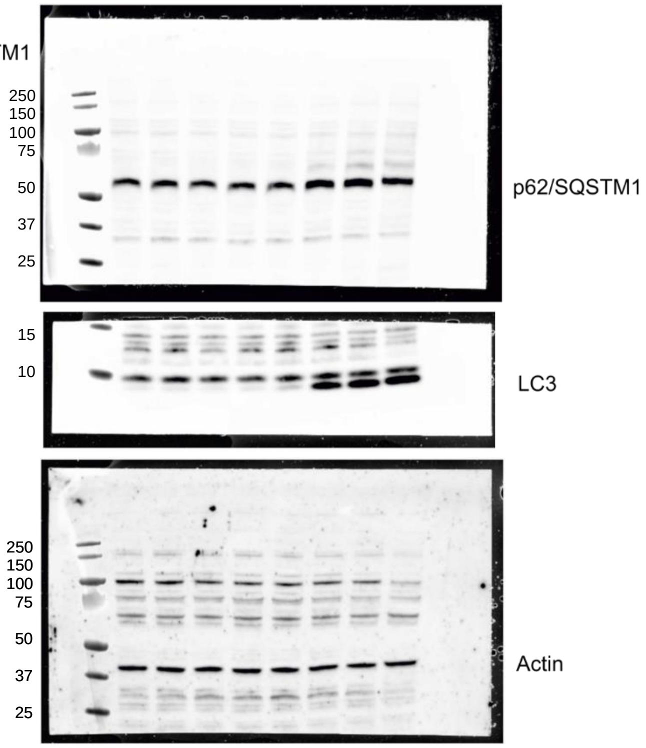

Supp. Fig. 1C

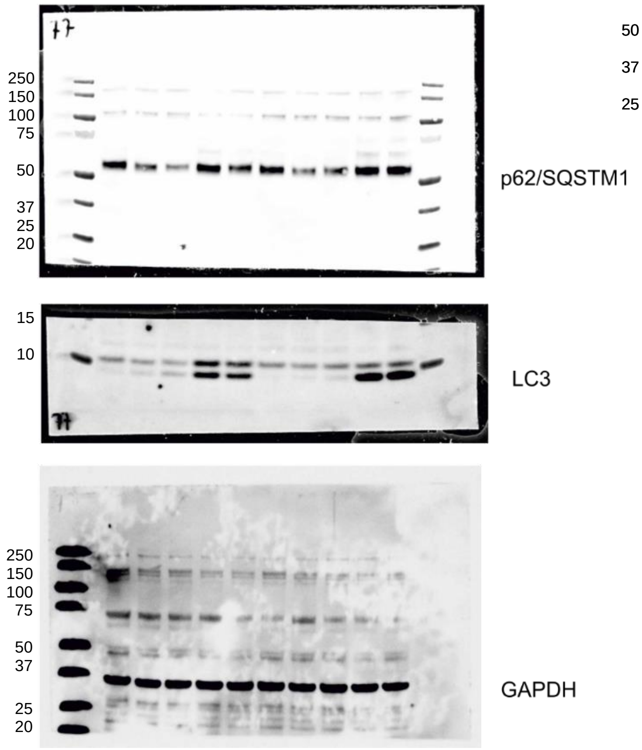

Supp. Fig. 1A

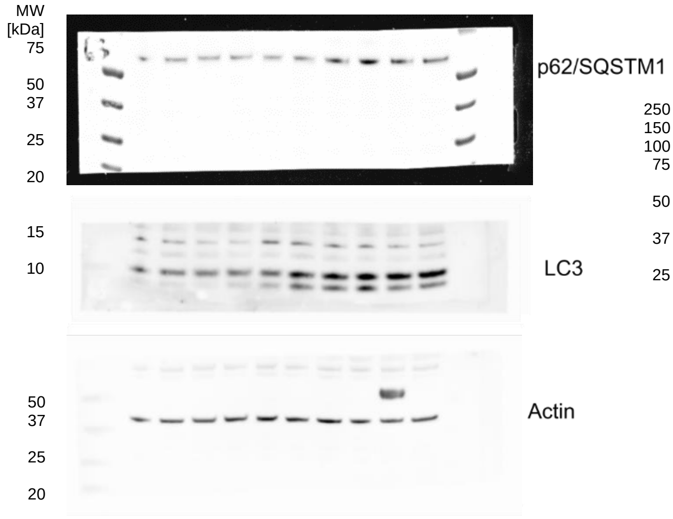

Supp. Fig. 1B

shorter exposures

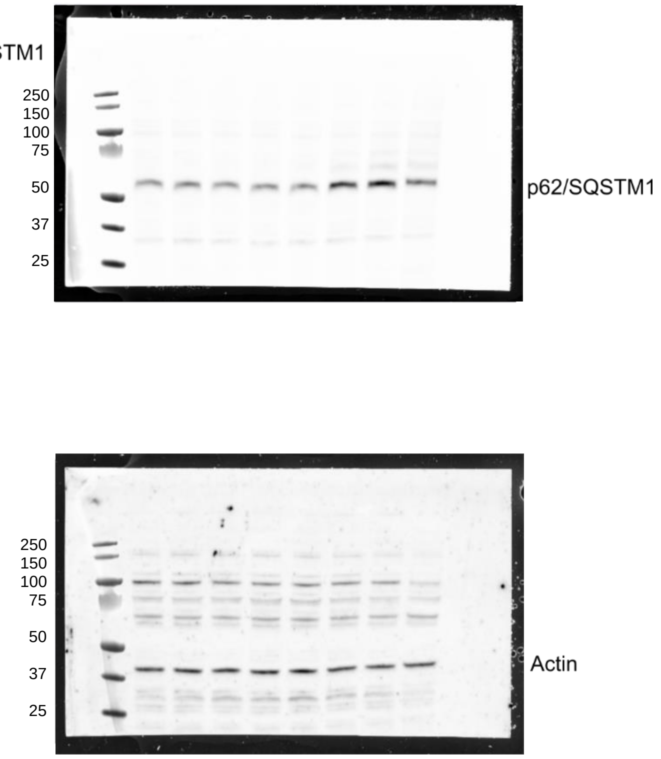

Supp. Fig. 1C

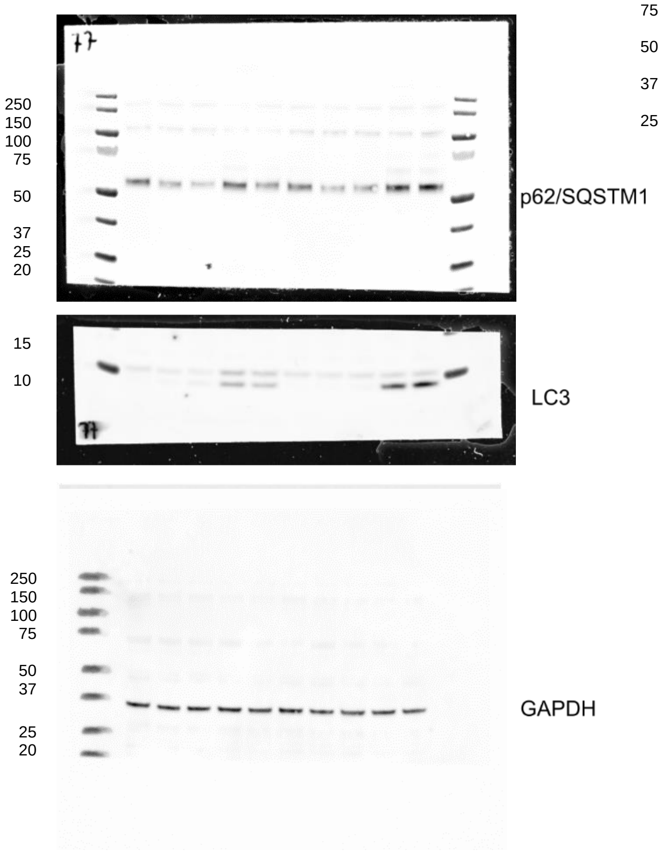

Supp. Fig. 3A

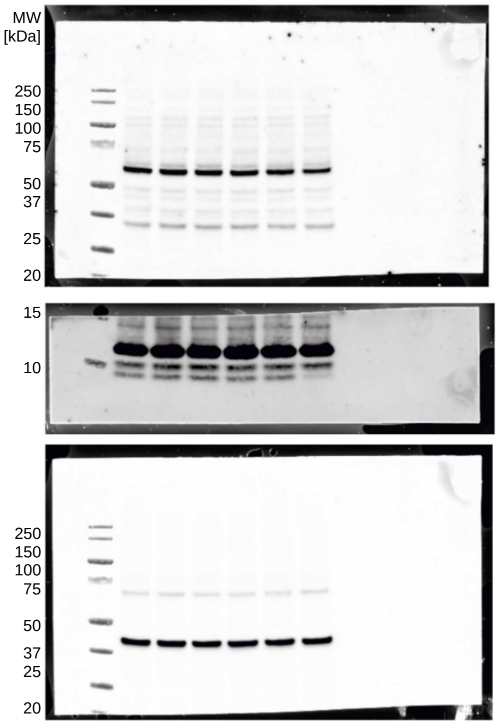

Supp. Fig. 3B

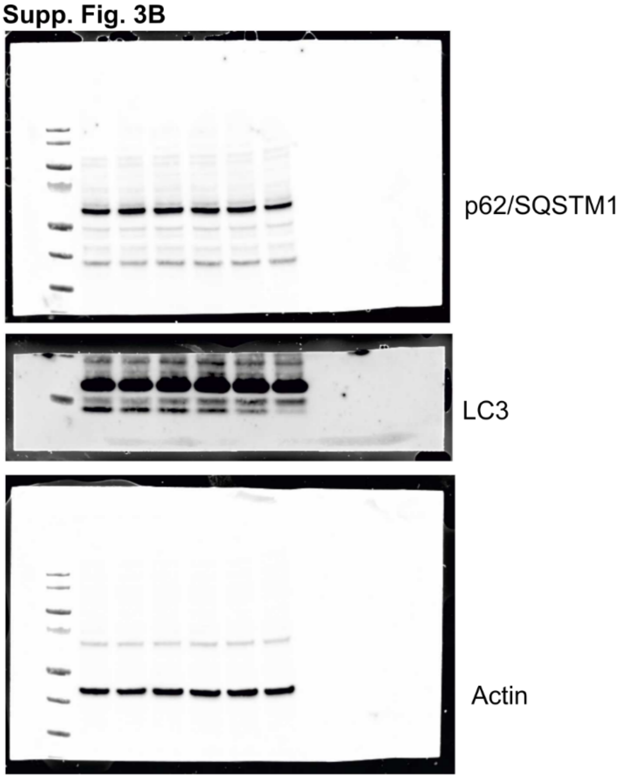

Supp. Fig. 3C

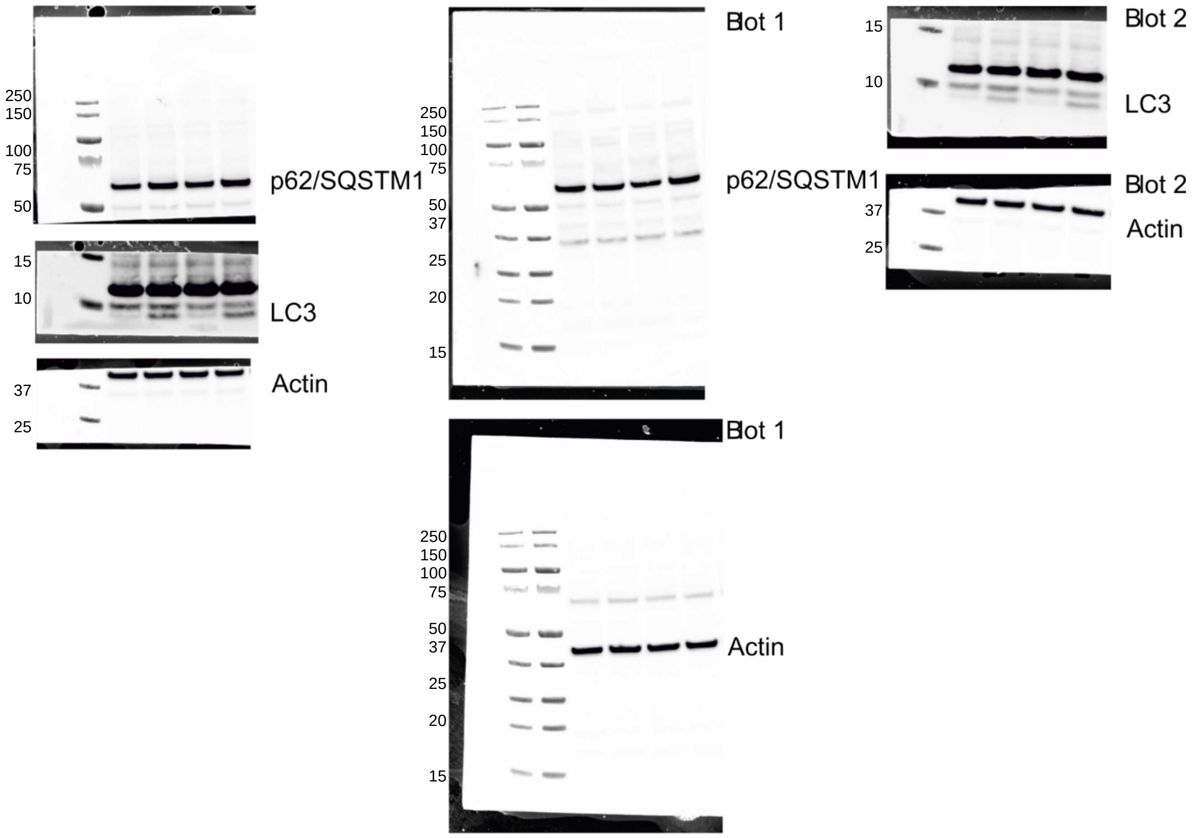

Supp. Fig. 3A

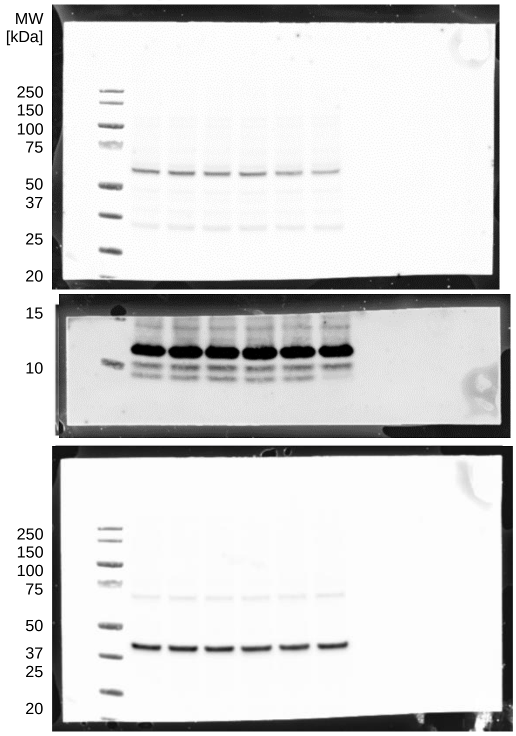

Supp. Fig. 3B

shorter exposures

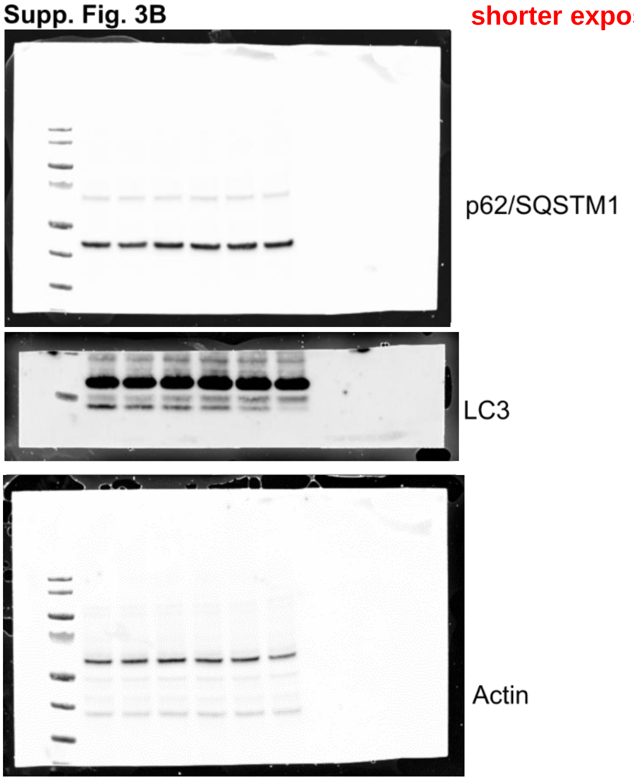

Supp. Fig. 3C

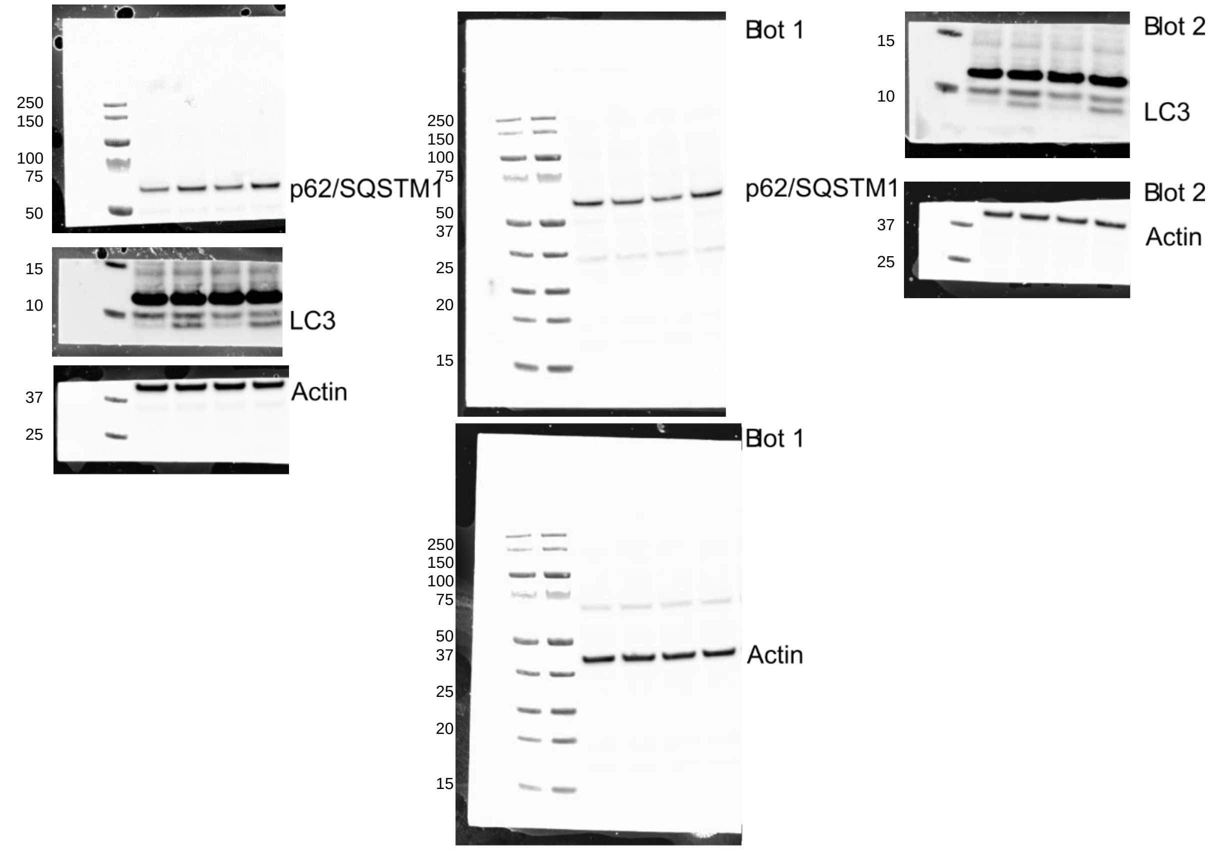

Supp. Fig. 3D

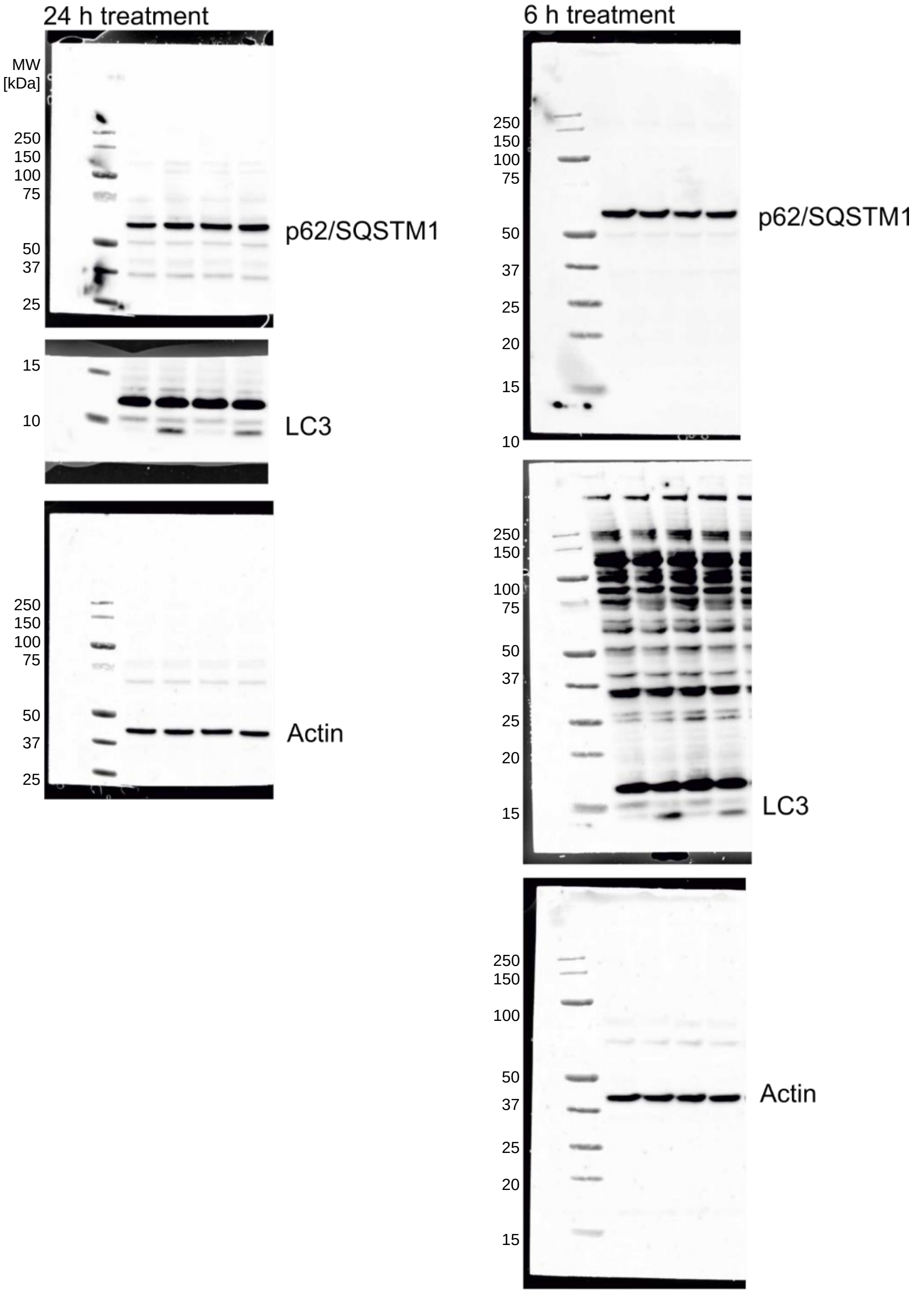
24 h treatment
MW [kDa]
250
150
100
75
50
37
25
p62/SQSTM1
15
10
LC3
250
150
100
75
50
37
25
Actin
6 h treatment
250
150
100
75
50
37
25
20
15
10
p62/SQSTM1
250
150
100
75
50
37
25
20
15
LC3
250
150
100
50
37
25
20
15
Actin

Supp. Fig. 3D

shorter exposures

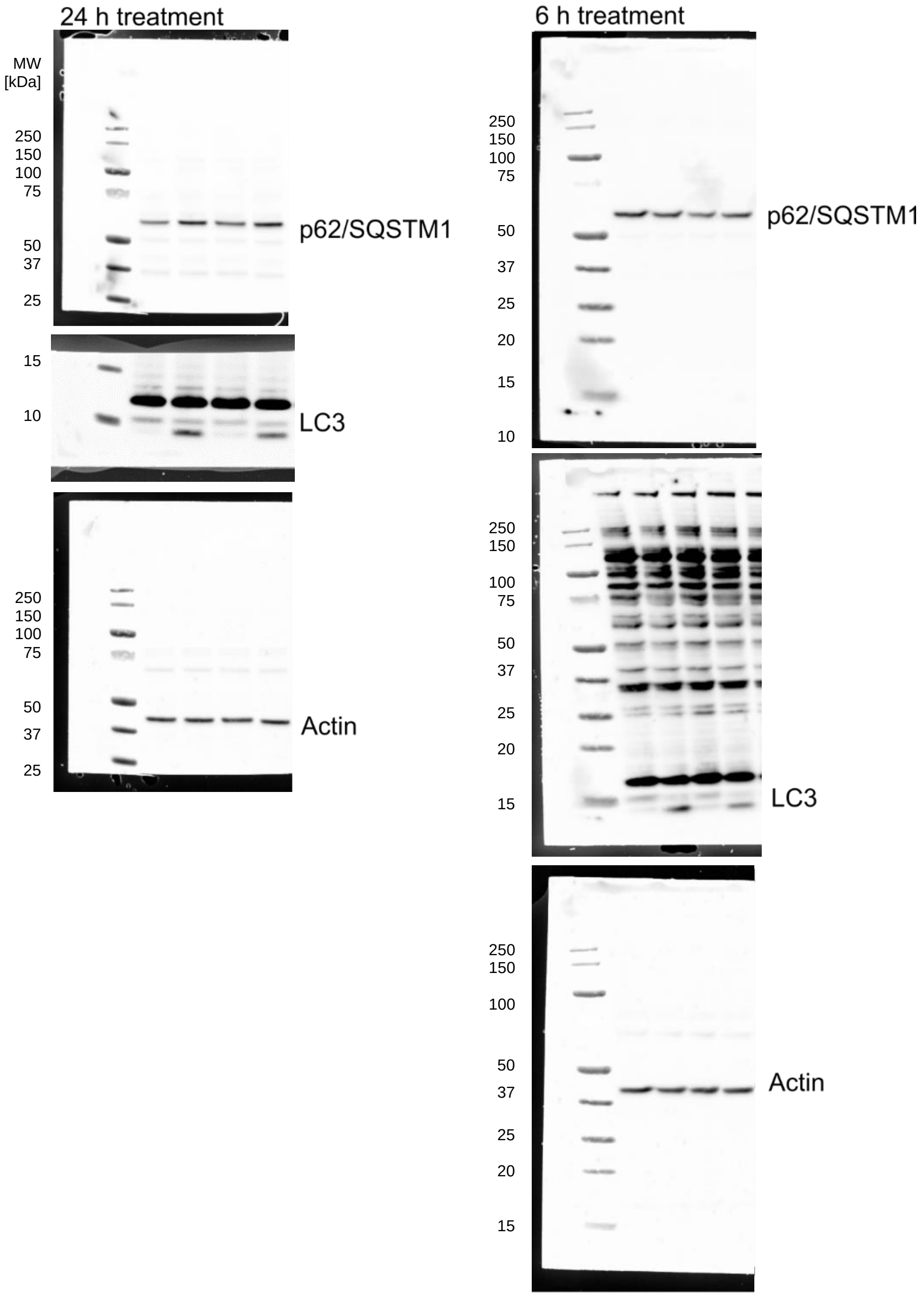

Supplement: Supplementary file 1 — Supplementary Information. [file 41598_2021_3875_MOESM1_ESM.pdf]
